# Transcriptional memory of gene expression across generations participates in transgenerational plasticity of field pennycress in response to cadmium stress

**DOI:** 10.3389/fpls.2022.953794

**Published:** 2022-09-30

**Authors:** Gengyun Li, Yuewan Zhao, Fei Liu, Minnuo Shi, Yabin Guan, Ticao Zhang, Fangqing Zhao, Qin Qiao, Yupeng Geng

**Affiliations:** ^1^College of Horticulture and Landscape, Yunnan Agricultural University, Kunming, China; ^2^Yunnan Key Laboratory of Plant Reproductive Adaptation and Evolutionary Ecology and Centre for Invasion Biology, Institute of Biodiversity, School of Ecology and Environmental Science, Yunnan University, Kunming, China; ^3^College of Chinese Material Medica, Yunnan University of Chinese Medicine, Kunming, China; ^4^Beijing Institute of Life Sciences, Chinese Academy of Sciences, Beijing, China; ^5^School of Agriculture, Yunnan University, Kunming, China

**Keywords:** field pennycress, phenotypic plasticity, maternal effect, transgenerational plasticity, transcriptional memory

## Abstract

Transgenerational plasticity (TGP) occurs when maternal environments influence the expression of traits in offspring, and in some cases may increase fitness of offspring and have evolutionary significance. However, little is known about the extent of maternal environment influence on gene expression of offspring, and its relationship with trait variations across generations. In this study, we examined TGP in the traits and gene expression of field pennycress (*Thlaspi arvense*) in response to cadmium (Cd) stress. In the first generation, along with the increase of soil Cd concentration, the total biomass, individual height, and number of seeds significantly decreased, whereas time to flowering, superoxide dismutase (SOD) activity, and content of reduced glutathione significantly increased. Among these traits, only SOD activity showed a significant effect of TGP; the offspring of Cd-treated individuals maintained high SOD activity in the absence of Cd stress. According to the results of RNA sequencing and bioinformatic analysis, 10,028 transcripts were identified as Cd-responsive genes. Among them, only 401 were identified as transcriptional memory genes (TMGs) that maintained the same expression pattern under normal conditions in the second generation as in Cd-treated parents in the first generation. These genes mainly participated in Cd tolerance-related processes such as response to oxidative stress, cell wall biogenesis, and the abscisic acid signaling pathways. The results of weighted correlation network analysis showed that modules correlated with SOD activity recruited more TMGs than modules correlated with other traits. The SOD-coding gene *CSD2* was found in one of the modules correlated with SOD activity. Furthermore, several TMGs co-expressed with *CSD2* were hub genes that were highly connected to other nodes and critical to the network’s topology; therefore, recruitment of TMGs in offspring was potentially related to TGP. These findings indicated that, across generations, transcriptional memory of gene expression played an important role in TGP. Moreover, these results provided new insights into the trait evolution processes mediated by phenotypic plasticity.

## Introduction

Phenotypic plasticity is the ability of organisms to change their phenotypes in response to environmental stimuli (Schlichting, 1986; Sultan, 2000; Pigliucci, 2005). Within-generation plasticity (WGP) allows organisms to match their traits to the local environment conditions (Colicchio and Herman, 2020). Moreover, plasticity may also occur between generations, termed as maternal effects or transgenerational plasticity (TGP), which accounts for the influences of parental environment (PE) on offspring development and phenotype (Agrawal et al., 1999; Galloway and Etterson, 2007; Bell and Hellmann, 2019). As the ecological and evolutionary significance of the phenotypic plasticity was realized and gradually elucidated (Kelly et al., 2012; Forsman, 2015), more attention has been paid to adaptive TGP because it can transfer potential ecologically and evolutionarily meaningful variation from parent to offspring in organisms (Herman and Sultan, 2011). When growth conditions are stable, the sessile lifestyle of plants and spatially limited dispersal of propagules usually make the PE a good predictor of the offspring’s environment (Sandner et al., 2018; Colicchio and Herman, 2020). As a result, the offspring’s fitness can be enhanced through adaptive TGP by shaping their phenotypes much earlier during development (Galloway and Etterson, 2007; Vu et al., 2015; Colicchio and Herman, 2020). Several studies demonstrated that TGP provides a pathway of adaptation that can influence the dynamics of selection and promote ecological breadth by maintaining populations under stressful environments (Sultan et al., 2009; Dyer et al., 2010; Herman et al., 2012). Therefore, TGP is widely believed to play an important role in plant adaptation and evolution in a challenging world (Lachmann and Jablonka, 1996; Kuijper et al., 2014; Leimar and McNamara, 2015; Bonduriansky, 2021).

Although increasing evidence has shown that TGP is common in nature (reviewed in Herman and Sultan, 2011; Donelson et al., 2017; Bonduriansky, 2021), the mechanisms responsible for the effects of PEs on offspring phenotypes remain elusive (Salinas et al., 2013; Herman and Sultan, 2016). Typically, phenotype is a result of genotype-by-environment interaction (G × E) (Des Marais et al., 2013), and the ability of phenotypic plasticity may be influenced by epigenetic regulation (Geng et al., 2013; Kooke et al., 2015). The cross-generational transmission of environmental cues may be mediated by epigenetic mechanisms and/or molecules that are packaged into seeds and transmitted from parents to offspring (Herman and Sultan, 2011). Epigenetic mechanisms, including small RNAs (sRNAs), DNA methylation, and histone modification, could regulate gene expression in offspring at transcriptional or post-transcriptional levels (Lang-Mladek et al., 2010; Pieterse, 2012; Quadrana and Colot, 2016). For example, DNA methylation induced by maternal environment for a part of sites in genome can be inherited stably by successive generations through sexual and/or asexual reproduction (Quadrana and Colot, 2016; Shi et al., 2019), thus affecting gene expression over multiple generations (Herman and Sultan, 2011; Fitz-James and Cavalli, 2022). Moreover, other maternally derived molecules, including mRNAs, proteins, phytohormones, and some primary and secondary metabolites, may also have effects on offspring traits (Verhoeven et al., 2010; Herman and Sultan, 2011). Although these mechanisms can independently or jointly influence the occurrence of TGP, all of them are ultimately functionalized by regulation of gene expression during offspring development (Bonduriansky, 2021). As a result, expression or transcriptional memory of selective genes could be one of the most important determinants of plasticity of gene expression. As gene expression connects environmental cues and trait expression, detection of changes in gene expression across generations can be used to identify biological processes and pathways underlying TGP, and to elucidate the molecular basis of TGP (Laitinen and Nikoloski, 2019; Sommer, 2020).

Recent studies mainly focused on inheritance of phenotypic trait changes and epigenetic memories in TGP (e.g., Herman and Sultan, 2016; Colicchio and Herman, 2020; Sobral et al., 2021). Although some researchers have begun to explore genomic reaction norms represented by plasticity of gene expression underlying the TGP process, knowledge about the extent to which gene expression is affected by PE is still limited (Rivera et al., 2021). Colicchio et al. (2015) first reported effects of PE on gene expression plasticity in offspring by discovering patterns of gene expression plasticity in response to parental leaf damage in *Mimulus guttatus*. In the same year, another study discovered the effects of parental-experienced salinity stress on expression of stored seed transcripts in *Medicago truncatula* (Vu et al., 2015). Some additional studies also revealed patterns of selective gene expression and transcriptional memory underlying the TGP process; however, most of these studies focused on animals (e.g., Clark et al., 2019; Shi et al., 2020). More evidence is needed to show a link between TGP and the plasticity or transcriptional memory of gene expression, especially in some widely distributed plants that are excellent at adapting to heterogeneous environments. Furthermore, it is well accepted that different genes usually work together as a complicated module and are simultaneously recruited in development to achieve a phenotype (Uller et al., 2018). However, knowledge about how co-regulated genes are recruited in the TGP process is still limited.

Field pennycress (*Thlaspi arvense* L.) is a diploid plant (2n = 2 × = 14) of Brassicaceae and widely distributes in temperate regions of the northern hemisphere (Best and McIntyre, 1975; Warwick et al., 2002; An et al., 2015). Field pennycress is commonly used as a biofuel to extract oil from seeds or as a cover crop to reduce soil erosion (Mitich, 1996; Sedbrook et al., 2014; Dorn et al., 2015; McGinn et al., 2019). It can also be eaten as a vegetable or used in traditional Chinese medicine. The genome of field pennycress was sequenced and assembled in our previous study, and was determined to have a genome of 527.3 Mb encoding 31,596 genes (Geng et al., 2021). Moreover, the results of an experiment that focused on transgenerational changes and dynamics of DNA methylation in response to abiotic stress, such as salinity in field pennycress, showed that the epigenetic diversity at the population level significantly increased, and some of the epi-alleles showed that transient epigenetic memory lasted at least two generations in offspring in non-stressed environment (Geng et al., 2020). It suggested that field pennycress might have some degree of TGP under environmental stress. Moreover, field pennycress mainly reproduces by self-pollination and produces seeds in less than 10 weeks (Warwick et al., 2002; Geng et al., 2021). These features make field pennycress an ideal for exploring the molecular basis underlying TGP as it is operable to control the genetic background and generate different generations in a short period.

Cadmium (Cd) is a metallic element with high pro-oxidant potential that is toxic to nearly all organisms (Kinraide, 2009). Last decades have witnessed serious Cd contamination in agricultural systems caused by increasing anthropogenic activities and industrialization (Cai et al., 2019). Some studies revealed that field pennycress is sensitive to Cd stress, and a series of plastic trait changes were identified in field pennycress response to Cd stress, including reduction in root length and shoot fresh weight, and decrease in leaf osmolality and membrane permeability (Monteiro and Soares, 2021). However, an ecotype that exhibited considerable Cd tolerance was reported in the Cd-polluted urban area of Jena, Germany, with higher activities of antioxidant enzymes and efficiency in excluding Cd compared with the normal ecotype (Martin et al., 2012). It can be inferred that TGP may participate in evolution of normal ecotypes of field pennycress in response to persistent Cd stress. Adaptive TGP is widely believed to be a fitness-enhancing strategy in similar environments across generations, thus it might play an important role in ecological adaptation and evolutionary processes (Bonduriansky, 2021). Analyses focusing on TGP and corresponding transcriptional memory of gene expression in response to Cd stress across different generations are useful for determining the role TGP plays in processes of plant adaptation and evolution under the persistence of environmental selection pressure.

This study aims to examine the pattern of trait reaction norms and its relationship with the corresponding co-regulation pattern of gene expression underlying TGP of field pennycress in response to Cd stress. We measured morphological traits (e.g., individual height, and biomass), reproductive traits (e.g., flowering time, number, and hundred grain weight of seeds), and Cd tolerance related physiological traits [e.g., content of reduced glutathione (GSH) and activity of superoxide dismutase (SOD), malondialdehyde (MDA) and photosystem II activity (Fv/Fm)] under control conditions and Cd treatment in parent individuals and offspring. The gene expression profiles in samples of different generations were also examined at the same time by RNA sequencing. This design allowed us to: (1) detect and compare reaction norms that represent WGP and TGP in response to increased soil Cd; (2) identify genes and pathways that play important roles in Cd stress adaptation in *T. arvense*; and (3) discover expression patterns of key genes that showed transcriptional memory in offspring, and assess their topological role in the recruitment of co-regulated gene modules underpinning TGP of field pennycress in response to Cd stress.

## Materials and methods

### Plant material and cadmium treatment

**FIGURE 1:**
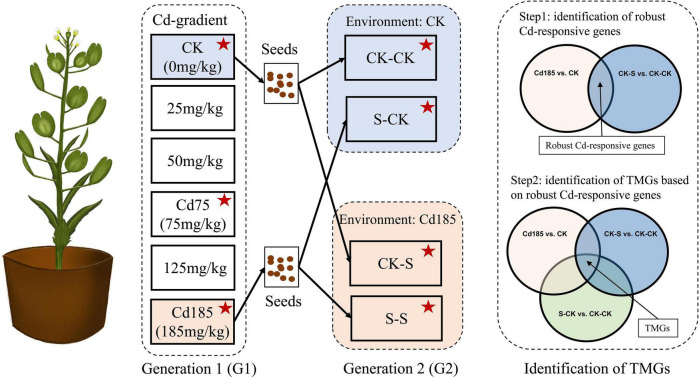
Experimental design. In G1, seedlings from maternal plant were treated with a gradient of Cd from 0 to 185 mg/kg. Seeds collected from CK and Cd185 were used to conduct experiment in G2 that treated offspring seedlings with CK and Cd185. Several morphological traits, reproductive traits and physiological traits of all individuals were measured, and samples marked by red star were further used for RNA sequencing. Transcriptional memory genes (TMGs) were identified by screened genes that showed the same expression pattern (both upregulated or downregulated) between Cd185 vs. CK, CK-S vs. CK-CK, and S-CK vs. CK-CK simultaneously.

Two generations of field pennycress were used in this study. For generation 1 (G1), seedlings were germinated from seeds of individuals collected from a wild population in Kunming (KM, alt. 1,910 m, N 30.313°, E 99.358°). The maternal plant was maintained in the greenhouse at Yunnan University, Kunming, for more than 2 years to eliminate wild environmental effects on seedlings used in experiment. After the first two new leaves appeared, the seedlings were individually transplanted into pots (9.5 cm in diameter, 7.5 cm in depth), and each pot contained 200 g of nutrition soil. CdCl_2_ solution was mixed with nutritious soil before transplant to conduct Cd treatment, with concentrations of 0 (CK), 25, 50, 75 (Cd75), 125, and 185 mg/kg (Cd185), for six Cd-treatment gradients (Figure 1). The G1 individuals were grown during July and September in 2020 with approximately 12 h of daytime and harvested in the middle of September.

To determine TGP, seeds collected from Cd 185 and CK individuals in G1 were used to germinate generation 2 (G2) seedlings. A total of four treatments were set for G2 individuals (Figure 1): (1) CK-CK: offspring of individuals in G1-CK treatment, continued growth in pots with the CK environment; (2) CK-S: offspring of individuals in G1-CK treatment, growth in pots with Cd185 treatment; (3) S-CK: offspring of individuals in G1-Cd185 treatment transplanted to pots with the CK environment; (4) S-S: offspring of individuals in G1-Cd185 treatment, continued growth in pots with Cd185 treatment. Individuals of G2 were grown from November to the following January in 2021, and the ambient temperature and illumination conditions were similar to those of G1 using compensation equipment for heating and lighting.

### Trait measurement

The following traits were measured for G1 and G2 individuals for at least six replications. For morphological traits: (1) The total biomass was determined based on the dry weight of the plant. (2) The individual height was measured by the length from the top to the end of stem. For reproductive traits: (3) The flowering time was defined as the number of days it took for the first flower to bloom. (4) The seed traits included the number of seeds and hundred-grain weight of seeds. For physiological traits: (5) The photosystem II activity was measured as Fv/Fm, which is the ratio between light-induced variable and maximum fluorescence of chlorophyll, using a pulse-amplitude modulated fluorometer (Junior-PAM, Walz GmbH, Effeltrich, Germany). (6) The SOD activity in leaves was determined by the SOD reagent box (A001-1, Nanjing Jiancheng, Nanjing, China). (7) The content of reduced GSH was determined using a CheKine reduced GSH colorimetric assay kit (Abbkine, Wuhan, China). (8) The level of lipid peroxidation (measured as MDA) in leaves was determined using a lipid peroxidation MDA assay kit (Abbkine, Wuhan, China). All measurements of physiological traits were determined according to the manufacturers’ instructions. Tukey’s HSD was used to test for significant differences between different treatments. A two-way ANOVA was used to test the effects of G1 PE, G2 offspring environment (OE), and their interactions.

### Transcriptome sequencing and data analysis

**FIGURE 2:**
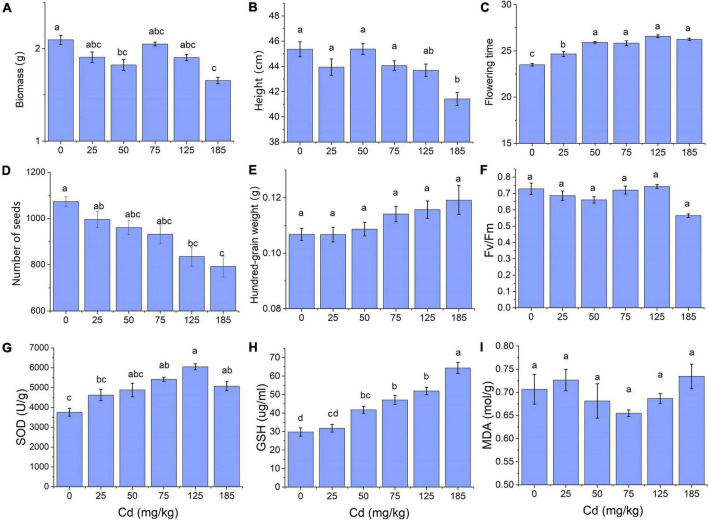
Trait expression of field pennycress in response to different soil Cd concentrations. **(A)** Biomass; **(B)** individual height; **(C)** flowering days; **(D)** number of seeds; **(E)** hundred-grain weight; **(F)** Fv/Fm; **(G)** SOD; **(H)** GSH; and **(I)** MDA. Quoted values are means ± SE (*N* = 6). Different letters indicate significant differences among groups determined by Tukey’s HSD.

According to the results of traits measurement under different concentrations of Cd stress, we found most of these traits showed significant differences under 185 mg/kg of Cd (Figures 2A,B). Thus we selected 185 mg/kg as an effective and intense concentration of Cd stress to induce morphological trait changes, and 125 mg/kg, 75 mg/kg were considered as a moderate and slight concentration, respectively. Finally, samples of CK, Cd75, and Cd185 in G1, and CK-CK, CK-S, S-CK, S-S in G2 were used to conduct RNA sequencing. For each treatment, three independent biological replications of each treatment were randomly selected. Total RNA of young and mature leaves was isolated using TRIzol reagent (Invitrogen, Carlsbad, CA, USA), and then equally mixed for each sample. RNA quality assessment was conducted using an Agilent 2100 Bioanalyzer (Agilent Technologies, Santa Clara, CA, USA). High-quality RNAs were then used for RNA sequencing by Novogene Bioinformatics Technology Co., Ltd, Beijing, China. Briefly, a standard library construction process, including fragmentation, first and second strand cDNA synthesis, adaptor ligation, PCR, and quality assessment, was conducted using purified mRNA from total RNA. After clustering process, a total of 21 libraries [(3 G1 treatments × 3 biological replicates) + (4 G2 treatments × 3 biological replicates)] were then sequenced on an Illumina Novaseq platform to generate 150-bp paired-end reads.

Clean data were obtained by removing reads containing adapter, ploy-N, and low-quality reads from raw data. Paired-end clean reads were aligned to the field pennycress reference genome (Geng et al., 2021) using Hisat2 v2.0.5. The mapped reads of each sample were assembled by StringTie (v1.3.3b), and featureCounts v1.5.0-p3 was used to count the number of reads mapped to each gene to calculate FPKM. In addition to genome annotation, transcripts were annotated to the TAIR10 database by BLAST.

Differential expression analysis was conducted based on FPKM using DESeq2 R package (1.20.0). Different standards for absolute log_2_ fold change for differentially expressed genes (DEGs) varied between studies, normally in a range of 0.3–2.0 (e.g., Wang et al., 2017, Montaldo et al., 2020). Genes with an adjusted absolute *p*-value < 0.05 in comparisons were identified, and log_2_ fold change was used to evaluate the degree of expression difference, with an absolute value above 0.5 assigned as significantly differentially expressed (Liu and Sun, 2021). According to this criterion, the Cd-responsive genes were identified by Cd75 vs. CK, Cd185 vs. Cd75, and CK-S vs. CK-CK. Some Cd-responsive genes enhanced their expression with increased Cd concentration, and these genes were identified by comparing Cd185 vs. Cd75.

To determine the transcriptional memory across generations, Cd-responsive genes that showed across generational persistent expression even if growth condition changed to normal in G2, were defined as “transcriptional memory genes” (TMGs). TMGs were identified by screened genes that showed the same expression pattern simultaneously in Cd185 vs. CK, CK-S vs. CK-CK, and S-CK vs. CK-CK. Briefly, we first screened genes with the similar expression pattern (both upregulated or downregulated) between Cd185 vs. CK and CK-S vs. CK-CK. This step was used to find robust Cd-responsive genes that can be induced or repressed by Cd stress across generations. The results were then cross-referenced with S-CK vs. CK-CK to select robust Cd-responsive genes with the same expression pattern in absence of Cd stress in G2. As a result, the intersection part was considered as TMGs (Figure 1). We further examined the expression enhancement of TMGs under the condition of consistent Cd stress across generations, and this was done by comparing expression pattern of TMGs with that under S-S vs. CK-S. The Gene ontology enrichment for Cd responsive genes was compared and visualized using WEGO 2.0 (Ye et al., 2018) and GO terms exhibiting a corrected *p*-value of ≤0.05 were considered significantly enriched.

### Gene co-expression network analysis

Gene co-expression network analysis was conducted using the WGCNA R package (Langfelder and Horvath, 2008). Briefly, FPKM data of Cd-responsive genes were normalized using the limma R package, and genes with too many missing entries were filtered using goodSamplesGenes function. The function pickSoftThreshold was then used to calculate scale free topology for multiple soft thresholding powers. According to the result, the unsigned network was constructed by setting a power of 16, minModuleSize of 30, and a mergeCutHeight of 0.25. Eigengenes for each module were calculated by using moduleEigengenes function with default parameters. Pearson’s correlation coefficient (PCC) of the module eigengenes to trait data was then assessed to identify key modules involved in Cd response and plasticity of transgenerational traits. After network construction, the top 10% of weighted edges were analyzed because millions of edges were generated. The module hub genes were identified by nodes with the top 5% degree in each module.

In order to examine whether the modules were robust and reproducible, the module preservation statistics were computed using the modulePreservation function implemented in WGCNA (Langfelder et al., 2011). The multiExpr was set by G1 and G2 samples to test module preservation between generations, with G1 as reference, and the number of permutations was set to 200. The computation generated a Zsummary and a medianRank for each module. For values of Zsummary, 2 > *Z* represented no preservation, 2 < *Z* < 10 represented weak to moderate preservation, and *Z* > 10 represented strong preservation. Module with lower median rank exhibit higher observed preservation statistics than a module with a higher median rank (Sharma et al., 2018).

### Quantitative real-time PCR verification of transcriptional memory genes

To validate the expression pattern of TMGs between generations, 1.0 μg of total RNA of each sample was reverse transcribed into cDNA using a PrimeScript™ RT reagent kit (TaKaRa, Dalian, China). The quantitative real-time PCR (qRT-PCR) was performed using TB Green™ Premix Ex Taq™ II (TaKaRa, Dalian, China) and the Roche LightCycler 96 system (Roche, Penzberg, Germany) following the manufacturers’ instructions. *UBQ10* was selected as the internal reference gene. All reactions were performed in triplicate for each sample, and the relative changes for comparison between different treatments were quantified using the 2^–ΔΔCt^ method as described in a previous study (Li et al., 2017). Primers are listed in Supplementary Table 1.

## Results

### Plastic responses of field pennycress to cadmium treatment in generation 1

For morphological traits measured in G1, total biomass and individual height were similar in Cd treatment of 0–125 mg/kg but significantly decreased in 185 mg/kg Cd treatment (*p* < 0.01, Figures 2A,B). The flowering time was significantly increased in 0–50 mg/kg Cd treatment (*p* < 0.01), and this was maintained in a higher concentration of soil Cd (Figure 2C). Seeds production decreased along with the increase of Cd concentration, and individuals exposed to Cd185 treatment produced fewer seeds compared with those exposed to 0 (*p* < 0.01) and 25 mg/kg (*p* = 0.03) (Figure 2D). For the physiological traits, total SOD and GSH significantly increased with the increase of soil Cd concentration (*p* = 0.036 and *p* < 0.01 for SOD and GSH, respectively; Figures 2G,H).

### Effects of generation 1 parental environment on generation 2 offspring traits

**FIGURE 3:**
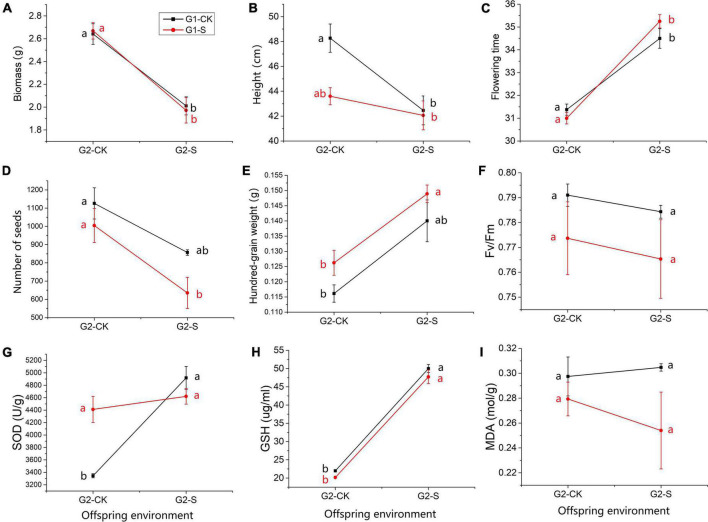
Reaction norm of trait expression in G2 individuals exposed to soil Cd concentrations of 0 mg/kg (CK) and 185 mg/kg (S). The red and black lines show seedlings that originated from offspring of Cd185 (S) and CK treatment in G1, respectively. **(A)** Biomass; **(B)** individual height; **(C)** flowering days; **(D)** number of seeds; **(E)** hundred-grain weight; **(F)** Fv/Fm; **(G)** SOD; **(H)** GSH; and **(I)** MDA. Quoted values are means ± SE (*N* = 6). Different letters indicate significant differences among groups determined by Tukey’s HSD.

Several traits in G2 showed similar changes in direction along with the change of environmental conditions (Figure 3). For example, biomass, flowering days, Number of seeds, hundred-grain weight, Fv/Fm, GSH and MDA didn’t show significant difference for CK-CK vs. S-CK and CK-S vs. S-S with Tukey’s test (*p*-value > 0.05) (Supplementary Table 7), and presented similar reaction norm in all offsprings (Figures 3A,C–F,H,I), indicating that PE in G1 did not affect the performance of offspring in G2 under different OEs. The individual height showed values that close to significant difference between comparison of CK-CK and S-CK (*p* = 0.056 in Tukey’s HSD test, Figure 3B). The SOD is the only trait that significantly differed between CK-CK and S-CK (*p* = 0.02 in Tukey’s HSD test, Figure 3G), ANOVA showed the significant effect of PE-by-OE interaction can only be detected on SOD (*p* < 0.01, Table 1).

**TABLE 1:** Effects of the parental environment (PE), offspring environment (OE), and their interactions on traits of field pennycress in generation 2.

	Source of variation					
Trait	*F* for PE	*P*-value for PE	*F* for OE	*P*-value for OE	*F* for PE × OE	*P*-value for PE × OE
Biomass	0.003	NS	48.386	<0.001	0.124	NS
Individual height	4.625	0.047	9.720	0.007	3.280	NS (0.089)
Flowering days	0.310	NS	120.034	<0.001	2.793	NS
Number of seeds	3.997	NS (0.063)	13.856	0.002	0.341	NS
Hundred-grain weight	3.592	NS (0.076)	21.671	<0.001	0.015	NS
Fv/Fm	1.793	NS	0.306	NS	0.004	NS
GSH	2.300	NS	424.886	<0.001	0.028	NS
SOD	4.192	NS (0.075)	22.450	0.001	13.153	0.007
MDA	2.270	NS	0.157	NS	0.509	NS

NS means no significant difference (*p* > 0.05).

### RNA sequencing of field pennycress in response to cadmium treatment

For each library, an average of 6.55 G of clean data was generated. The average error rate was 0.02, the average Q30 was 94.88%, and the GC contents ranged from 45.62 to 47.99% (Supplementary Table 2). A total of 95.49 to 98.26% of clean reads were mapped to the field pennycress reference genome, which had been deposited in the National Center for Biotechnology Information (NCBI) with BioProject accession number PRJNA715950 (Geng et al., 2021), with the unique mapping rate ranging from 90.67 to 95.90% for each library (Supplementary Table 3). A total of 86.76 to 90.14% of clean reads were mapped to exon regions, and approximately 2.31 to 2.91% of clean reads were mapped to intron regions (Supplementary Table 4). Different biological replicates showed a high correlation with average squared PCC of 0.93. Finally, 33061 transcripts were identified according to the mapping result. The high quality of sequencing data allowed subsequent analysis.

### Genomic reaction norm in response to cadmium treatment

**FIGURE 4:**
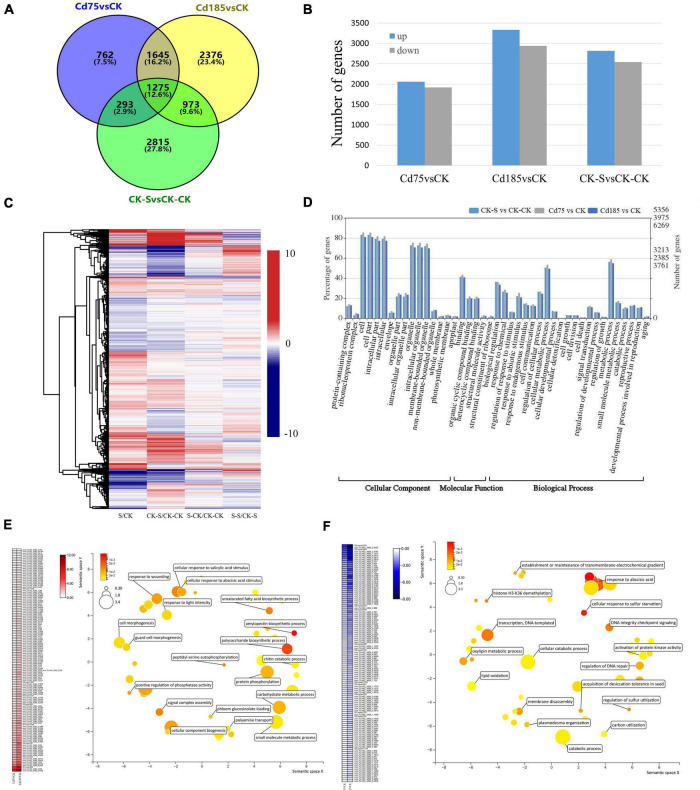
Expression and function of Cd-responsive genes. **(A)** Comparison and distribution of Cd-responsive genes in G1 (Cd75, Cd185) and G2 (CK-S) using CK (G1) and CK-CK (G2) as controls. **(B)** Numbers of upregulated and downregulated genes. **(C)** Differential expression patterns of Cd-responsive genes. The color represents log_2_ fold change values; negative values (blue) represent downregulation and positive values (red) represent upregulation. **(D)** Gene Ontology (GO) classifications of the DEGs. **(E,F)** Expression and biological processes of genes with enhanced upregulation **(E)** or downregulation **(F)** along with the increase of soil Cd concentration in G1. For the heatmap, color was adjusted to log_2_ fold change of comparison pairs. For GO map results, the color and size were adjusted to *p*-value and log_10_ *p*-value, respectively.

The Cd-responsive genes were identified by three comparison pairs of Cd-treated samples vs. control, including Cd75 vs. CK, Cd185 vs. CK, and CK-S vs. CK-CK. A total of 3,975, 6,269, and 5,356 Cd-responsive genes were identified in the Cd75, Cd185, and CK-S libraries, respectively. The results showed that high concentrations (Cd185, and CK-S) of Cd treatment led to more responsive genes than low concentrations (Cd75). After merging results, a total of 10,028 Cd-responsive genes were obtained (Figure 4A). Among them, only 762 (7.50%) genes were exclusively included in Cd75 vs. CK, indicating that these genes only participate in slight (Cd75) Cd stress, while the rest of them can be detected in both slight and intense (Cd185) Cd stress (3213 genes, 31.7%), or only participate in intense Cd stress (6,164 genes, 60.8%) (Figure 4A). However, there are only 2,248 genes identified to be differentially expressed in both G1 (Cd185 vs. CK) and G2 (CK-S vs. CK-CK) (Figure 4A), indicating that these genes can be induced differential expression robustly by Cd-stress in both G1 and G2, and differential expression of other genes (4,021 in G1 and 3,108 in G2) maybe caused by batch effects or some other stochastic factors rather than Cd-stress. The numbers of upregulated genes were 2,058, 3,331, and 2,815 for Cd75 vs. CK, Cd185 vs. CK, and CK-S vs. CK-CK, respectively (Figure 4B). The expression patterns of Cd-responsive DEGs are shown in Figure 4C.

The GO enrichment analysis showed that these DEGs participated in biological processes such as response to environmental stimulus, regulation of growth and developmental processes, cellular detoxification, and reproductive processes (Figure 4D). Furthermore, 131 and 118 genes had enhanced upregulated or downregulation along with the increased concentration of soil Cd (Figures 4E,F). These upregulated genes were enriched in cellular response to salicylic acid and abscisic stimulus, unsaturated fatty acid biosynthetic process, and protein phosphorylation etc.; while the downregulated genes were enriched in histone H3-K36 demethylation, cellular response to sulfur starvation, and oxylipin metabolic process etc. (Figures 4E,F) in the GO enrichment analysis.

### Cadmium responsive transcriptional memory in generation 2 individuals

**FIGURE 5:**
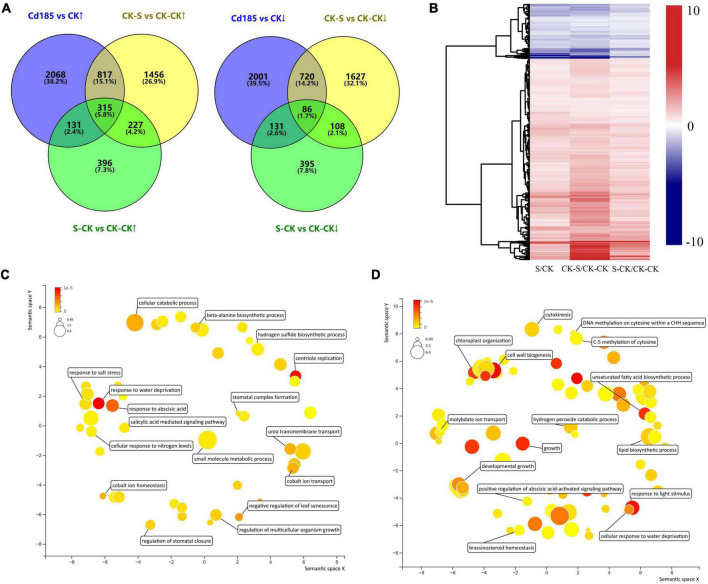
Identification and characterization of Cd-responsive TMGs. **(A)** Comparison and distribution of upregulated and downregulated genes in Cd185 (G1), CK-S (G2), and S-CK (G2) using CK (G1) and CK-CK (G2) as controls. Genes with intersection were identified as TMGs. **(B)** Heatmap showing the expression profile of TMGs. The color scale represents log_2_ fold change between different comparison pairs. **(C,D)** Biological processes of downregulated **(C)** and upregulated **(D)** TMGs. Representative biological processes are labeled; the color and size were adjusted to *p*-value and log_10_ *p*-value, respectively.

The TMGs were identified by screening genes that showed the same expression pattern (both upregulated and downregulated) in intersection of three comparison pairs (Cd185 vs. CK, CK-S vs. CK-CK, and S-CK vs. CK-CK). A total of 315 upregulated and 86 downregulated TMGs were identified (Figure 5A and Supplementary Table 5), with the expression pattern shown in Figure 5B. The GO enrichment analysis showed that TMGs participated in some important biological processes. For example, the downregulated TMGs were involved in biological processes such as response to abscisic acid (ABA), the salicylic acid-mediated signaling pathway, and regulation of multicellular organism growth (Figure 5C), whereas the upregulated TMGs participated in the biological processes of cell wall biogenesis, response to light stimulus, the hydrogen peroxide catabolic process, and DNA methylation of cytosine within a CHH sequence (Figure 5D).

According to expression pattern, the TMGs were further compared with the S-S vs. CK-S results to identify the TMGs that may enhance expression under the S-S environment when G1 and G2 both experienced Cd treatment. As a result, a total of 17 TMGs (7 downregulated and 10 upregulated) that showed enhancement of expression under the S-S environment were identified, and the detailed information was provided in Supplementary Table 6.

### Gene co-expression modules related to cadmium stress adaptation and measured traits in leaves

A total of 18 modules were identified based on pairwise correlations of gene expression across all samples (Supplementary Figure 1). The results of module preservation statistics showed that four modules (gray, grey60, tan, and salmon) were not preserved as their Zsummary below 2 and medianRank above 12, five modules (red, green, yellow, midnight blue, and lightcyan) showed a weak to moderate preservation as their Zsummary range from 3.4 to 9.8 and medianRank range from 1 to 17. The rest of modules showed a good module preservation (Supplementary Figure 2).

**FIGURE 6:**
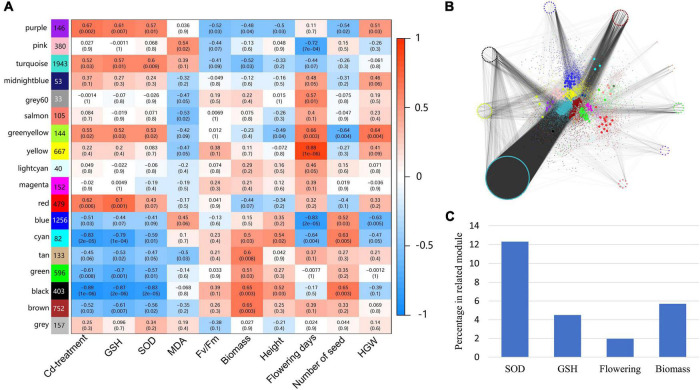
Correlation of traits and modules, and distribution of TMGs in different modules. **(A)** Module–trait associations. The module eigengenes and traits are represented in rows and columns, respectively. The corresponding correlation (represented by value and color) and *p*-value are contained in each cell. Number beside module name indicate count of genes in this module. **(B)** A global view of TMGs distributed in each module. Modules are represented by different colors, and key hub genes are represented by larger circles. The TMGs are surrounded on the outside. **(C)** Proportion of TMGs in trait-correlated modules (PCC > 0.6 and *p* < 0.05). More TMGs were found in SOD-related modules than those of other traits.

Because only leaf samples were used in RNA sequencing, traits of other organs, including individual height, number of seeds, and hundred-grain weight, were not considered in the module-trait associations analysis. As flowers were modified leaves, flower primordia may be included when collecting young leaf samples; thus, flowering days were included in the analysis. Results of module-trait associations analysis showed that modules related to Cd-treatment also tend to associate with GSH and SOD (Figure 6A). According to the criterion of *p*-value < 0.05, four modules (purple, turquoise, greenyellow and red) were positively related to Cd-treatment, GSH and SOD, five modules (cyan, tan, green, black and brown) were positively related to biomass, and three modules (gray 60, greenyellow and yellow) were positively related to flowering days (Figure 6A).

Transcriptional memory genes were found to be dispersed into different modules. Among them, most TMGs were assigned to the turquoise (228 TMGs out of 1821 nodes), brown (29 out of 702), black (27 out of 251), and greenyellow modules (26 out of 118), and the remaining modules included fewer than 20 TMGs (Figure 6B). The percentage of TMGs in trait-related modules with PCC > 0.6 was calculated, and the results showed that more TMGs were found in SOD-related modules than in other traits (Figure 6C).

**FIGURE 7:**
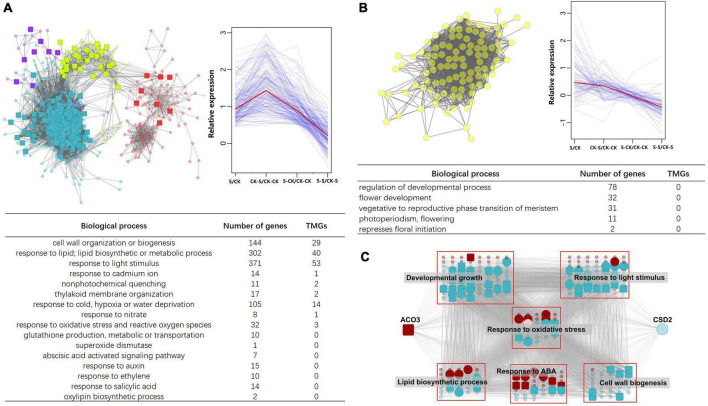
The TMGs in trait-related modules. **(A)** TMGs that participate in Cd stress adaptation and are co-expressed with other important genes in a sub-network of four modules (turquoise, red, greenyellow, and purple) that are correlated with Cd treatment, GSH, and SOD. Pictures showing the expression pattern of TMGs and its co-expression relationship with other genes. Function and corresponding gene numbers are listed at the bottom. **(B)** Gene co-expression relationship, expression pattern, and function in the yellow module, which is highly correlated with flowering time. No TMGs were identified in this module. **(C)** Sub-network formed by *ACO3*, *CSD2*, and their neighbors. Different nodes are classified according to gene function. For simplicity, neighbors of the other functions are not shown. Key hub genes are represented by larger circles, TMGs are represented by large squares, and modules that the gene belongs to are represented by different colors.

To discover the influences of TMGs on topological structure and overall function of gene co-expression modules in response to Cd treatment, four modules (purple, turquoise, greenyellow, and red) that positively correlated with Cd treatment, GSH, and SOD were focused on. Several upregulated TMGs and other functional related genes were included in these modules. Functional analysis showed module genes participate in biological processes such as cell wall organization or biogenesis, lipid biosynthesis and metabolism, response to Cd, and other abiotic stimuli (Figure 7A). Among them, some TMGs were identified as hub genes. For example, harbinger transposon-derived protein 2 (*HDP2*) (Ta.Chr.1.1011) is a transcription factor of the trihelix family. According to the annotation, this gene showed a function in prevention of DNA hypermethylation and epigenetic silencing. Moreover, *HDP2* is co-expressed with the most genes (a total of 1,365 nodes) in the turquoise module, indicating its important role in the topological construction of this key module. By contrast, analysis of flowering day-related modules showed that, although the module had a large number of genes associated with flower development, no TMGS existed in this module (Figure 7B). This result may somehow be correlated with no transgenerational plastic memory in flowering day traits.

Although transgenerational memory was detected in SOD activity, we failed to detect transcriptional memory in copper/zinc superoxide dismutase 2 (*CSD2*) (Ta.Chr.3.2310), the unique superoxide dismutase coding gene in the turquoise module. However, ACONITASE 3 (*ACO3*) (Ta.Chr.5.1524), which interplays with *CSD2*, was found to be one of the TMGs in the brown module. We isolated *ACO3*, *CSD2*, and their neighbors from the global network; as a result, a sub-network was formed (Figure 7C). Like the sub-network in Figure 7A, this sub-network also consisted of several TMGs and other functions-related genes that participate in biological processes including developmental growth, response to light stimulus, lipid biosynthesis, response to ABA, cell wall biogenesis, response to oxidative stress, and other processes (Figure 7C). Furthermore, the sub-network of *ACO3*-*CSD2*-neighbors included a total of 110 TMGs, of which 57 (51.82%) were hub genes according to the criteria of the top 5% of degree in each module.

### Validation of the expression pattern of transcriptional memory genes by quantitative real-time PCR

**FIGURE 8:**
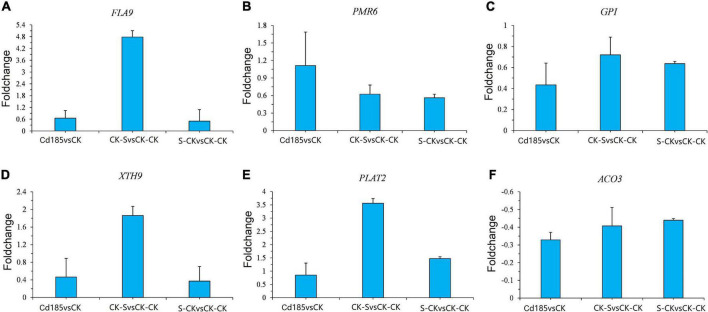
Validation of expression pattern of TMGs by qRT-PCR. **(A–F)** qRT-PCR results for six randomly selected TMGs. The *y*-axis represents the log_2_ ratios between comparison pairs along the *x*-axis. Relative expression quantification was carried out using the 2^– ΔΔCt^ method, with UBQ10 as the internal reference gene. Error bars represent mean ± SE (*n* = 3).

For validation of RNA sequencing accuracy, the expression of six TMGs was analyzed through qRT-PCR, as shown in Figure 8. The relative expressions of Cd185 vs. CK, CK-S vs. CK-CK, and S-CK vs. CK-CK were calculated using the qRT-PCR results. The results showed a similar pattern to RNA-seq. For example, for the cell wall organization gene powdery mildew resistant 6 (*PMR6*) (Ta.Chr.2.4195), the results of qRT-PCR showed the same pattern as fold change calculated by RNA-seq data (Log2 fold change value of 1.49, 2.47 and 1.18 for Cd185 vs. CK, CK-S vs. CK-CK, and S-CK vs. CK-CK, respectively), both of them showed an upregulated pattern (Figure 8B). These results demonstrate the accuracy of RNA-seq in this study.

## Discussion

### Within-generation and transgenerational plasticity in field pennycress

Phenotypic plasticity is common in nature and essential for organisms surviving in a changing world (Kelly et al., 2012; Forsman, 2015), especially for plants with a sessile lifestyle. In this study, we examined within-generation and TGP in field pennycress in response to different concentrations of soil Cd. In the parental generation (G1), a series of phenotypic changes were observed along with increasing Cd concentration, including a decrease in individual height and biomass, longer time to flowering, and enhancement of SOD activity and content of GSH (Figure 2). These phenotypic variations are not necessarily adaptive. For example, the decreases in individual height and biomass in Cd-treated individuals are the same as the results of another study that conducted similar Cd treatment on field pennycress (Monteiro and Soares, 2021); this indicates that growth inhibition by Cd is universal between different genotypes. However, growth inhibition was not observed under the same conditions in the congener *Thlaspi praecox*, which is a hyperaccumulator that is highly tolerant to Cd stress (Tolra et al., 2006; Monteiro and Soares, 2021).

In contrast, enhancement of SOD activity and content of GSH may be useful to increase fitness under long-term Cd stress. The main mechanism of Cd phytotoxicity is the induction of oxidative stress (Loix et al., 2017). When plants deal with oxidative stress, SOD constitutes the first line of defense against ROS because it is involved in scavenging and detoxification of ROS (Alscher et al., 2002; Mishra and Sharma, 2019). Moreover, GSH participates in metal homeostasis, antioxidative defense, and signal transduction under metal-induced oxidative stress (Jozefczak et al., 2012). It was clear that SOD activity and content of GSH were increased in response to high concentrations of soil Cd, these increases may be beneficial to field pennycress to maintain normal physiological function and protect cells from injury during oxidative stress.

Furthermore, TGP was found for SOD activity in offspring. A significant parental-by-offspring interaction (PE × OE) was identified in SOD activity (Table 1). The offspring derived from G1-S-treated plants maintained higher SOD activity, even when grown under normal conditions in G2 (S-CK), compared with offspring derived from growth under normal conditions in G1 and G2 (CK-CK) (Figure 3G). For species growing in sufficiently stable environments, many lines of evidence have confirmed that effects of TGP can be adaptive when parent and progeny environments match (Walsh et al., 2016; Colicchio and Herman, 2020; Halali and Saastamoinen, 2022). In other words, inheritance of adaptive trait changes through TGP can lead to an increase of fitness in offspring when parent and progeny environments match or can be expected to be similar (Sandner et al., 2018; Colicchio and Herman, 2020). Limited ability for seed dispersal in field pennycress makes growth conditions highly predictable across generations. Therefore, transgenerational maintenance of SOD activity may be considered as adaptive for offspring growth under Cd-polluted environment in which future stress could be predicted according to the environment of maternal plants.

On the contrary, according to the theory of limit and cost of phenotypic plasticity, the expression of plastic traits may lead to impairment in fitness if there is a mismatch between final phenotype and environment (DeWitt et al., 1998; Relyea, 2002; Murren et al., 2015). TGP of SOD activity may not be adaptive for individuals whose habitat is converted to a normal condition, which is inaccurately predicted by the maternal environment. However, the consequence of adaptive TGP on SOD activity was not obvious in S-S compared with CK-S individuals. It can be inferred that differences in fitness may be detected in individuals derived from maternal plant growth under Cd stress for several generations rather than using those that were only treated for one generation.

### Transcriptional memory genes played important roles in adapting to cadmium stress in *Thlaspi arvense*

As a sort of phenotype at the molecular level, genomic reaction norm, which is represented by gene expression under different conditions, is useful for understanding mechanisms of phenotypic plasticity (Aubin-Horth and Renn, 2009). According to the results of Cd-responsive genes in G1, approximately 1.57 times as many DEGs were induced by a high concentration of soil Cd (Cd185) compared with a low concentration (Cd75), and some Cd-responsive genes showed a pattern of enhanced expression with the increase of Cd concentration (Figures 4E,F). These results indicated that genomic reaction norm and phenotypic reaction norm present non-random changes in direction and slope along with environmental gradient. Moreover, according to function analysis of Cd-responsive genes, some biological processes, including response to oxidative stress, cell wall biosynthesis, sulfur utilization, and ABA signal transduction, were also found to be enriched in other species in response to Cd stress, such as tall fescue (*Festuca arundinacea*), pakchoi (*Brassica chinensis* L.), and rice (*Oryza sativa* L.) (Zhou et al., 2016; Zhu et al., 2018; Sun et al., 2019). The results indicated that these processes might be universal in Cd responses of plants.

The screening criteria for TMGs is rigorous and based on intra- and intergenerational biological replicates. As a result, only approximately 4% of Cd-responsive genes were identified as TMGs. Some TMGs have been proven to play an important role in response to Cd stress in plants. For example, several upregulated TMGs, such as transcripts of NADPH-dependent thioredoxin reductase C (*NTRC*) (Ta.Chr.3.760) and 2-Cys peroxiredoxin A (*2CPA*) (Ta.Chr.5.3838), were found to be involved in the biological processes of superoxide radical removal and hydrogen peroxide catabolism, which are useful for ROS homeostasis and chloroplast protection against oxidative damage (Pérez-Ruiz et al., 2006; Zheng et al., 2020). The ABA signaling pathway plays an important role in Cd stress tolerance (Leng et al., 2021). Several genes involved in regulation of the ABA signaling pathway showed transcriptional memory of upregulation in Cd-treated maternal plants and offspring grown under normal condition, such as transcripts of receptor dead kinase 1 (*RDK1*) (Ta.Chr.1.701), tetratricopeptide-repeat thioredoxin-like 1 (*TTL1*) (Ta.Chr.4.2161), and LYSM-CONTAINING RECEPTOR-LIKE KINASE 3 (*LYK3*) (Ta.Chr.4.2285) (Rosado et al., 2006; Paparella et al., 2014; Kumar et al., 2017). Additionally, the cell wall is a barrier to prevent Cd from entering and damaging the protoplast. Several upregulated TMGs were involved in cell wall organization or biogenesis, such as xyloglucan endotransglucosylase/hydrolase 9 (*XTH9*) (Ta.Chr.0.3619), GPI-anchored protein (*GPI*) (Ta.Chr.5.3158), and PLAT DOMAIN PROTEIN 2 (*PLAT2*) (Ta.Chr.3.4335) (Kushwah et al., 2020; Depuydt and Vandepoele, 2021).

The expression patterns of some of these genes were examined by qRT-PCR (Figure 8). Furthermore, a DNA methyltransferase associated with maintenance of genome-wide DNA methylation, chromomethylase 2 (*CMT2*) (Ta.Chr.1.2289) (He et al., 2021), also showed transcriptional memory of upregulation in Cd-treated maternal plants and offspring grown under normal conditions. The transcription memory of these key Cd stress-responsive genes may influence the fitness of offspring under long-term Cd stress. Our previous study showed that some of the DNA methylation modification of epi-alleles caused by salinity stress can be inherited by at least two generations of offspring in non-stressed environments (Geng et al., 2020); thus, it can be inferred that epigenetic memory may contribute to the transcriptional memory in *T. arvense*. In addition to epigenetics, many other mechanisms contribute to transcriptional memory, such as transmission of mRNAs, proteins, and hormones from parent to offspring (Herman and Sultan, 2011). More experiments are needed to examine the causes of transcriptional memory in field pennycress in response to Cd stress.

### Recruitment of transcriptional memory genes in co-expression gene modules correlated with transgenerational plasticity

The recruitment of most TMGs by a limited number of modules reveals that co-regulation of TMGs may play an important role in the TGP of *T. arvense*. The superoxide dismutase coding gene *CSD2*, which was recruited in the turquoise module, was not identified as a TMG according to our criteria. However, we found that *CSD2* was co-expressed with many other oxidative stress-responsive genes, and several of these genes showed transcriptional memory (Figure 7C). *ACO3* encodes aconitase and is co-expressed with *CSD2*, and its expression pattern was validated by qRT-PCR (Figure 8F). Evidence showed that aconitase acts as a negative regulator that is specifically bound to 5′ UTR of the *Arabidopsis CSD2 in vitro*, an increased level of *CSD2* transcript was observed in aconitase-knockout plants, leading to more tolerance to oxidative stress in aconitase-knockout plants (Moeder et al., 2007).

In this study, *ACO3* was one of the TMGs that was downregulated in both G1 and G2 Cd-treated individuals, and also showed transcriptional memory in G2 S-CK individuals. Transcriptional memory of *ACO3* may indirectly affect SOD activity in G2 S-CK individuals, thus contributing to TGP of SOD activity. The sub-network constructed by *CSD2*, *ACO3*, and their neighbors included a considerable amount of hub TMGs, indicating recruitment of TMGs in offspring potentially related to TGP. In addition to *ACO3*, the other TMGs involved in biological processes, including lipid biosynthesis, cell wall biogenesis, response to ABA, developmental growth, and response to light stimulus, were co-expressed as neighbors of *ACO3* and *CSD2* (Figure 7C), some of them were proven to play important roles in Cd stress tolerance (Abd_Allah et al., 2015; Loix et al., 2017; Leng et al., 2021). This result indicates that these pathways may be associated with TGP of SOD activity in *T. arvense*. In contrast, there were no TMGs found in the yellow module, which was highly related to flowering time. Many genes that participate in flower development and transition from vegetative to reproductive phases were recruited in the yellow module; however, none of them were identified as TMGs or co-expressed with TMGs (Figure 7B). This may be one of the reasons why TGP was not observed for this trait.

The increase of SOD activity induced by TGP in offspring may be recognized as the first step in the process of genetic assimilation. Genetic assimilation is a phenomenon that describes the decrease of environmental sensitivity of gene expression, which may lead to fixed or constitutive expression of environmentally induced traits in the absence of a recurrent environmental cue (Waddington, 1961; Ehrenreich and Pfennig, 2016; Nijhout et al., 2021). It can be inferred from the results that high SOD activity maintained in offspring may ultimately be fixed if a high soil Cd concentration is maintained for many generations, just as in the Cd-tolerant field pennycress ecotype reported in the Cd-polluted urban area of Jena, Germany (Martin et al., 2012). Transcriptional memory, which is often neglected in research on TGP, may play an important role in the processes of trait evolution mediated by phenotypic plasticity.

## Data availability statement

The data presented in this study are deposited in the SRA database of NCBI repository, accession numbers: SRR18883184 – SRR18883204.

## Author contributions

GL performed the data analysis and drafted the manuscript. YZ, FL, MS, TZ, and YGu performed the wet lab work. FZ, QQ, and YGe participated in data analysis and design of the study. YGe conceived the idea, participated in the design of the study, and finalized the manuscript. All authors have read and approved the final manuscript.

## Supplementary material

Supplementary Figure 1 Gene co-expression network construction. **(A)** Clustering dendrogram of genes with the original module colors and assigned merged module colors. **(B)** Soft threshold of network construction. Power = 16 was selected.

Supplementary Figure 2 The medianRank preservation statistics **(A)** and the Zsummary preservation statistics **(B)** of the modules. The dashed blue and green lines indicate the thresholds of 2 < Zsummary < 10.
