# Supplementary figures and images for: Transcriptional memory of gene expression across generations participates in transgenerational plasticity of field pennycress in response to cadmium stress
Source: Front Plant Sci. 2022 Sep 30;13:953794. doi: 10.3389/fpls.2022.953794 (PMC9561902; doi:10.3389/fpls.2022.953794)

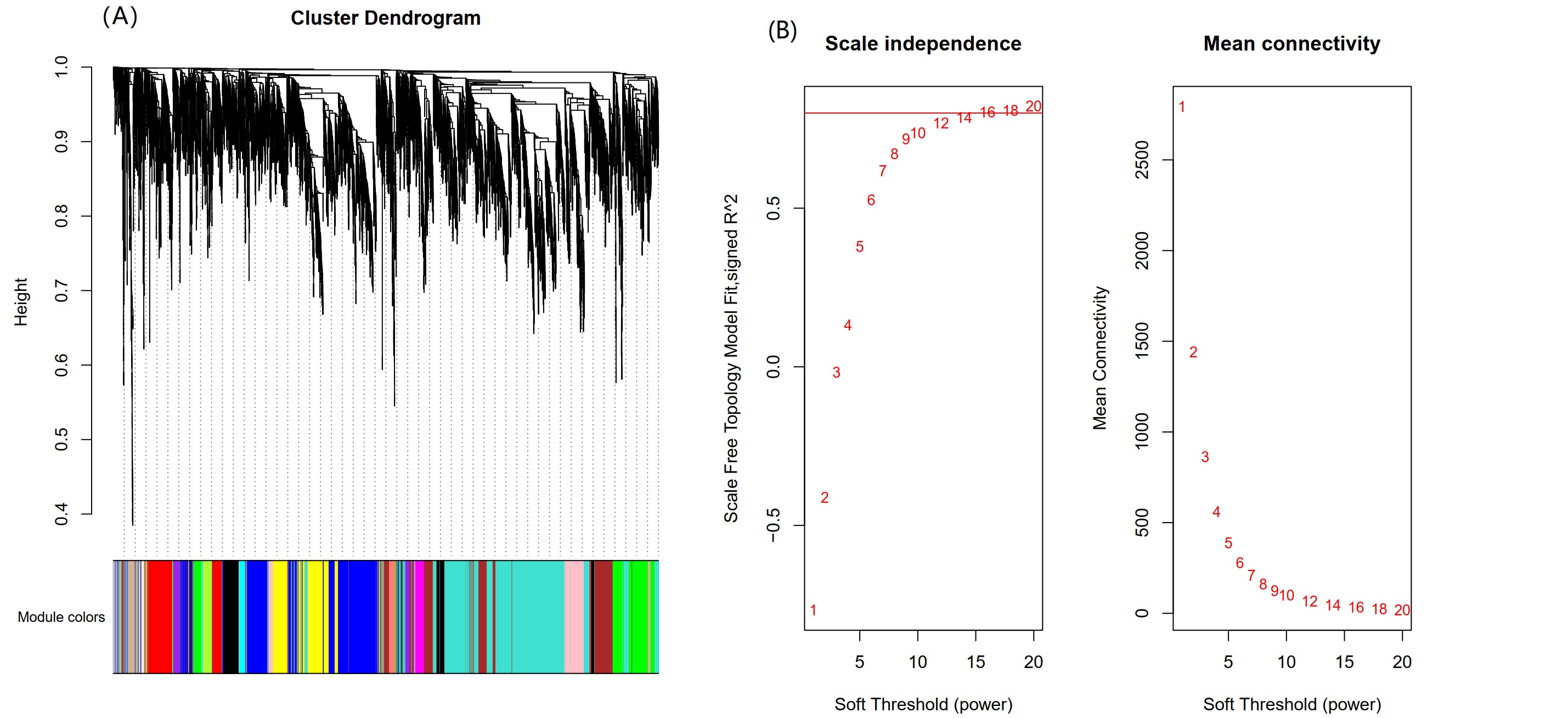

Supplement: Supplementary Figure 1 — Gene co-expression network construction. (A) Clustering dendrogram of genes with the original module colors and assigned merged module colors. (B) Soft threshold of network construction. Power = 16 was selected. [file Image_1.JPEG]

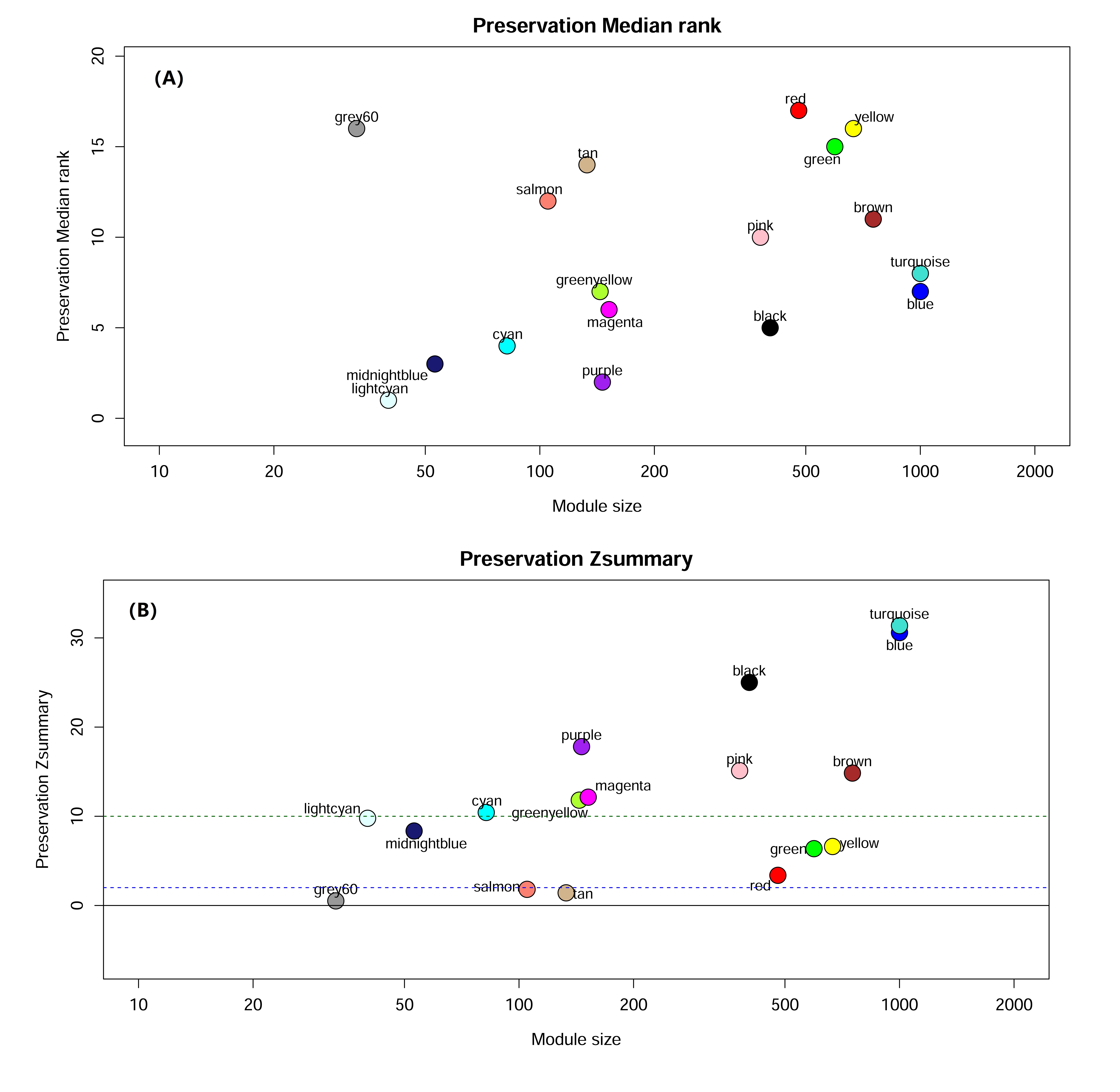

Supplement: Supplementary Figure 2 — The medianRank preservation statistics (A) and the Zsummary preservation statistics (B) of the modules. The dashed blue and green lines indicate the thresholds of 2 < Zsummary < 10. [file Image_2.JPEG]
